# Synthesis of sericin-based conjugates by click chemistry: enhancement of sunitinib bioavailability and cell membrane permeation

**DOI:** 10.1080/10717544.2016.1267822

**Published:** 2017-02-09

**Authors:** Luca Scrivano, Domenico Iacopetta, Maria Stefania Sinicropi, Carmela Saturnino, Pasquale Longo, Ortensia Ilaria Parisi, Francesco Puoci

**Affiliations:** 1 Department of Pharmacy, Health and Nutritional Sciences, University of Calabria, Rende, CS, Italy,; 2 Department of Sciences, University of Basilicata, Potenza, Italy, and; 3 Department of Chemistry and Biology, University of Salerno, Fisciano, SA, Italy

**Keywords:** Sericin, sunitinib, bioconjugates, radical grafting, bioavailability, cell permeability

## Abstract

Sericin is a natural protein that has been used in biomedical and pharmaceutical fields as raw material for polypeptide-based drug delivery systems (DDSs). In this paper, it has been employed as pharmaceutical biopolymer for the production of sunitinib–polypeptide conjugate. The synthesis has been carried out by simple click reaction in water, using the redox couple l-ascorbic acid/hydrogen peroxide as a free radical grafting initiator. The bioconjugate molecular weight (50 kDa < Mw < 75 kDa) was obtained by SDS-PAGE, while the spectroscopic characteristics have been studied in order to reveal the presence of grafted sunitinib. In both FT-IR and UV/Vis spectra, signals corresponding to sunitinib functional groups have been identified. Since sunitinib is an anticancer drug characterized by low bioavailability and low permeability, the bioconjugation aimed at their enhancement. *In vitro* studies demonstrated that bioavailability has been increased to almost 74%, compared with commercial formulation. Also cell membrane permeability has been augmented in *in vitro* tests, in which membrane models have been used to determine the lipid membrane/physiological fluid partition coefficient (Kp). The log(Kp) value of the bioconjugate was increased to over 4. This effect resulted in a three-fold decrease of IC_50_ value against MCF-7 cells.

## Introduction

Biopolymer–drug conjugates have recently attracted much attention as drug delivery systems (DDSs). Bioconjugates generally aim at the exploitation of the properties of the biopolymeric material at which the drug is attached (Kok et al., 1999). The scientific literature is rich of examples of drug–polymer conjugates: some of them are represented by chitosan–drug conjugates, in which the highly biocompatible and biodegradable natural occurring polysaccharide bears therapeutic molecules, like methotrexate, through covalent linkage (Fattahi et al., 2015), or by dextran–catechin grafted conjugates (Vittorio et al., 2012). Several studies demonstrated that conjugation of both natural or synthetic polymers (Etrych et al., 2012; Puoci et al., 2012) with small anticancer molecules could be carried out for the achievement of a more stable, but still endowed of intrinsic activity, drug-conjugate. Conjugation, usually, results in the production of stable DDSs characterized by long-term activity.

Besides, protein–drug conjugates, such as antibody–anticancer drug conjugates, optimize specificity and target the delivery of cytotoxic agents to the tumor. Thereby, targeting will affect the biodistribution of the drugs, sparing normal tissue exposure to the cytotoxic agent and allowing the use of potent agents that would cause high toxicity for systemic use (Alley et al., 2010; Akash et al., 2016).

For information about drug–polymer conjugates, a more detailed review of the literature can be found elsewhere (Vicent, 2007; Pang et al., 2013; Coburn & Kaplan, 2015).

In the present work, a bioconjugate was obtained *via* grafting of a model drug onto silk-derived protein sericin (SER), a by-product of textile industry. Silk filaments, produced by the silkworm *Bombyx mori*, are made of a double strand of fibroin, held together by a gummy coating mainly constituted by SER. During silk processing, the silk filaments are treated with boiling water. In this way, SER is removed and discarded (degumming).

Sericin’s molecular weight, ranging from about 10 to over 400 kDa, strongly depends on the extraction and isolation methods used. Molecular weight also influences the solubility in aqueous solutions: smaller polypeptides having a molecular weight less than 60 kDa are more soluble in cold water; on the other hand, SER fractions having molecular weight over 60 kDa are poorly soluble in water at room temperature, but still soluble in hot water (Zhang et al., 2006b).

The highly water-soluble SER is a biocompatible and biodegradable protein, easily hydrolyzed by proteolytic enzymes (Deng et al., 2014; Liu et al., 2015), which makes it a bioresorbable material useful for medical and pharmaceutical applications (Padamwar & Pawar, 2004; Lamboni et al., 2015; Parisi et al., 2015a). When used in combination with a drug, sericin is able to establish weak interactions that only slightly affect its secondary structure (Napavichayanun et al., 2015).

Currently, only a few examples of SER-based conjugates have been developed as DDSs. In 2006, Zhang et al. have prepared two SER bioconjugates. In a first report, a sericin–l-asparaginase conjugate for l-asparaginase modification and delivery was produced. The enzyme is, in fact, used as a chemotherapeutic agent in the management of acute lymphoblastic leukemia. The conjugation resulted in a greatly improved affinity between l-asparaginase and its substrate, l-asparagine (Zhang et al., 2006b). In the same year, a SER–insulin bioconjugate with improved biological stability was reported. SER–insulin conjugates were found to have a prolonged half-life compared to bovine serum albumin-insulin conjugates and native insulin (Zhang et al., 2006a). Immunogenicity and antigenicity were not observed for both conjugates in *in vivo* experiments. Thus, these authors demonstrated that SER bioconjugates can be efficiently applied as delivery systems.

In this paper, we report for the first time the conjugation of a synthetic drug to sericin. In this work, a small molecular tyrosine kinase inhibitor (sunitinib, SUT) has been chosen as model drug.

Small molecular tyrosine kinase inhibitors (smTKIs) are powerful anticancer drugs that are experiencing rapid growth. SmTKIs include imatinib, gefitinib, erlotinib, afatinib, dasatinib, bosutinib, ponatinib, etc., divided in first-, second- and third-generation TKIs (Jabbour et al., 2015).

Among smTKIs, SUT, a second-generation drug, is a multi-targeted receptor TKI orally administered for the treatment of gastrointestinal stromal tumors, advanced renal cell carcinomas and progressive, well-differentiated pancreatic neuroendocrine tumors (Wu et al., 2014; Parisi et al., 2015b). SUT possesses anti-cancer and anti-angiogenic activities, due to the potent inhibition of vascular endothelial growth factor receptors (types 1–3), platelet derived growth factor receptor (α and β), as well as fms-like tyrosine kinase 3, stem-cell factor receptor, colony-stimulating factor receptor (type 1) and glial cell-line derived neurotrophic factor receptor (Izzedine et al., 2007; Papaetis & Syrigos, 2009).

From a pharmacokinetic point of view, sunitinib is classified by the biopharmaceutics classification system (BCS) as a class IV drug (Herbrink et al., 2015). BCS establishes possible absorption-related issues for drugs, like SUT, characterized by low bioavailability. Drug solubility and cell permeability are, indeed, critical parameters that influence the absorption process, hence the bioavailability. BCS classifies drugs as: Case I: high solubility and high permeability; Case II: low solubility and high permeability; Case III: high solubility and low permeability; Case IV: low solubility and low permeability (Amidon et al., 1995). SUT indeed is very poorly soluble in water and ethanol, but highly soluble in DMSO (Kassem et al., 2012), thus the therapeutic effect of SUT might be limited in physiological aqueous media. In order to improve the solubility of SUT in aqueous solutions, conjugation with water soluble biopolymeric macromolecules is a valuable method.

With the purpose of improving its solubility and cell permeability, a sericin–sunitinib (SER–SUT) bioconjugate was obtained *via* free radical grafting of sunitinib onto sericin. An easy click reaction has been employed to carry out the synthesis. The product SER–SUT conjugate, has been studied by FT-IR and UV/Vis spectroscopy and SDS-PAGE. Bioavailability, membrane permeability and cytotoxic activity have been evaluated through *in vitro* models.

Conjugation with SER could be applied to a variety of drugs that are similar to SUT, such as bosutinib, crizotinib, nilotinib, vemurafenib among smTKIs, but also amphotericin B, chlorothiazide, colistin, ciprofloxacin, mebendazole, methotrexate, neomycin, furosemide, hydrochlorothiazide. They are all classified as Class IV drugs by BCS and possess similar properties to SUT (Wu et al., 2014, Herbrink et al., 2015).

## Methods

### Materials and instrumentations

Sunitinib malate, hydrogen peroxide (H_2_O_2_), l-ascorbic acid (AA), hydrochloric acid (37% w/w), disodium hydrogen phosphate, sodium dihydrogen phosphate, sodium hydrogen carbonate, pepsin from porcine gastric mucosa, esterase from porcine liver, α-amylase from porcine pancreas, pancreatin from porcine pancreas, sodium cholate, bile extract porcine and l-α-phosphatidylcholine from egg yolk were purchased by Sigma-Aldrich (Sigma Chemical Co., St. Louis, MO).

All solvents were reagent-grade or HPLC-grade and provided by Carlo Erba Reagents (Milan, Italy).

Dialysis tubes MWCO: 3500 Da and 12 000–14 000 Da were provided by Spectrum Laboratories Inc (Rancho Dominguez, CA).

IR spectra were recorded as films or KBr pellets on a Jasco FT-IR 4200 (Easton, MD). Absorption spectra were recorded with a Jasco V-530 UV/Vis spectrometer (Easton, MD).

### Sericin extraction

The water in which the silkworm cocoons are boiled during silk production has been provided by a local silk manufacturer. The water soluble fraction (sericin) dissolved in the solution was collected after centrifugation at 7000 rpm for 20 min. The light colored supernatant was then dialyzed for 72 h against distilled water using 3.5 kDa MWCO dialysis membrane. After 24 h of lyophilization, the sericin powder was obtained and used for further experimentations.

### *Sericin*–*sunitinib conjugate synthesis*

Single-step grafting of sunitinib onto sericin, by employing hydrogen peroxide/l-ascorbic acid as redox pair, was carried out as follows: in a suitable beaker 40 mL of water and 15 mL of ethanol were poured. 200 mg of sericin and 40 mg of sunitinib malate were dissolved in the reaction mixture. After complete dissolution, 2.5 mL of H_2_O_2_ (30% v/v) containing 83.5 mg of l-ascorbic acid were added. The mixture was maintained under magnetic stirring at 25 °C for 24 h under atmospheric pressure. The obtained SER–SUT conjugate was purified by dialysis (3.5 kDa MWCO) for 12 h against ethanol/water and for further 48 h against water. The solution recovered was then frozen and lyophilized to a powder.

### SDS-PAGE and silver staining

All chemicals used in SDS-PAGE and gel staining were obtained from Bio-Rad Laboratories S.r.l. or Sigma-Aldrich (Milan, Italy) and the solutions were prepared in deionized water. The protein samples were solubilized in loading buffer and gels were run on a Protean III mini-electrophoresis unit (BioRad, Milan, Italy) according to Laemmli (1970). After the run, gels were silver stained as reported elsewhere (Madeo et al., 2009; Zhao et al., 2012).

### *In vitro* bioavailability studies

The *in vitro* bioavailability study was carried out in a simulated gastric and intestinal environment through the previously reported method of the dialysis tubing procedure (Grande et al., 2016). The experiment is based on two successive enzymatic phases: pepsin and pancreatin digestions, which occurs in the first 2 h and in the following 4 h, respectively. The two phases are described as follow.

#### Pepsin digestion

30 mg of SER–SUT conjugate were put into a dialysis bag (MWCO 12–14 kDa) with 1.0 mL of a 0.85 N HCl solution, 3.0 mL of a sodium cholate solution (2% w/v in distilled water) and 24 000 U of porcine pepsin per mL. The dialysis bag was carefully sealed on each end with clamps and immersed in 10 mL of a 0.85 N HCl solution (pH 1.0). The system was then left for 2 h into a water-bath at 37 ± 0.5 °C.

#### Pancreatin digestion

After 2 h, the dialysis bag was recovered and carefully opened in order to allow the addition of 1.3 mL of a 0.8 M NaHCO_3_ solution, 11 mg of amylase, 11 mg of esterase and 11 mg of porcine pancreatin to the bag content. The dialysis bag was then sealed again and placed into 10 mL of a phosphate buffer saline (PBS) solution at pH 7.0. The incubation was repeated for further 4 h, into the shaking water bath at 37 ± 0.5 °C.

In order to evaluate the *in vitro* bioavailability of the sample, 3 mL of the medium used to mimic gastric and intestinal environment (0.85 N HCl solution pH 1.0 and PBS pH 7.0) were withdrawn at the time points of 2 and 6 h, respectively. The concentrations of the samples were determined by UV/Vis spectroscopy (at 426 nm) and calculated by using the equations obtained from the calibration curves of SUT standard solutions at pH 1.0 and 7.0, respectively. For this purpose, all the prepared standard solutions were analyzed by UV/Vis spectrophotometer and the correlation coefficient (R^2^) and slope of the regression equations obtained by the method of least square were calculated at pH 1.0 and 7.0, respectively.

The same procedure was applied to evaluate the bioavailability of sunitinib pharmaceutical formulation, commercially available under the name of Sutent®, used as control for data comparison. Each experiment was repeated three times.

## *In vitro* membrane permeability studies

### Liposomes preparation

Giant unilamellar vesicles (GUVs) were prepared according to the method reported by Moscho et al. (1996) and Walde et al. (2010). Briefly, 125 mg of PC were dissolved in 30 mL of a chloroform/methanol solution (1:2). 6.0 mL of the PC stock solution were put into a round bottom flask and 9.0 mL of PBS solution (pH 6.8) were gently poured along the flask walls. The organic solvents were removed using a rotary evaporator, under reduced pressure at 40 °C and 40 rpm. The GUVs suspension was transferred in a 10 mL volumetric flask and brought to volume with PBS solution. In order to produce small unilamellar vesciles (SUVs), the suspension was sonicated in an ice-water bath. Sonication was performed for 2 min and repeated 4 times with 2 min of interval.

SUVs size and distribution were determined by dynamic light scattering (DLS) analysis using 90 Plus particle size analyzer (Brookhaven Instruments Corporation, New York, NY) at 25.0 ± 0.1 °C by measuring the autocorrelation function at 90°. The laser was operating at 658 nm. The distribution size and the polydispersity index were directly obtained from the instrument.

### Determination of partition coefficient

SUVs obtained have been used as a cell membrane model to calculate the membrane/buffer solution partition coefficient of SER–SUT conjugate, using derivative spectrophotometry, as reported by Takegami et al. (2015).

9 sample solutions containing SUT (20 μM) and various amounts of the SUV suspension with a concentration of PC in the range of 0–1200 μM were prepared. The reference solutions were prepared without SUT. Each vial containing the sample was shaken and incubated for 2 h in order to reach the equilibrium state.

The absorption spectra were measured using 1 cm light-pass length cuvette, in the wavelength range between 400 and 550 nm with interval of 0.5 nm. The second-derivative spectra were calculated using “spectra manager” software v. 1.53.01, based on the Savitzky–Golay method in which the second-order polynomial convolution of 21 points was employed (Savitzky & Golay, 1964).

### Cell viability assay

Cell viability was determined using the 3-(4,5-dimethylthiazol-2-y1)-2,5-diphenyltetrazolium (MTT, Sigma–Aldrich, Milan, Italy) assay as reported by Saturnino et al. (2014) with some modifications. Briefly, MCF-7 cells were seeded on forty-eight well plates and grown in DMEM-F12 containing 10% fetal bovine serum (FBS), 1% l-Glutamine, 1 mg/ml penicillin–streptomycin. MCF-10A human mammary epithelial cells were cultured in DMEM-F12 supplemented with 10% horse serum (HS), 1% l-Glutamine, 1% penicillin/streptomycin, 0.5 mg/ml hydrocortisone, 20 ng/ml hEGF (human epidermal growth factor) and 0.1 mg/ml cholera enterotoxin (Sigma–Aldrich, Milan, Italy) and 10 μg/ml insulin. SUT was dissolved in dimethylsulfoxide (DMSO) (Sigma, St. Louis, MO) at a concentration of 50 mM and diluted in DMEM/F12 medium supplemented with 1% FBS (MCF-7) or 1% HS (MCF-10A) to obtain the working concentration. SER–SUT conjugated and SER were directly dissolved in DMEM/F12 medium supplemented with 1% FBS (MCF-7) or 1% HS (MCF-10A). Before the treatment, cells were serum deprived for 24 h, then treated with six different concentrations of SUT, SER–SUT or SER for 72 h in medium containing 1% FBS (MCF-7) or 1% HS (MCF-10A). At the end of the treatment, fresh MTT re-suspended in PBS was added to each well to a final concentration of 0.2 mg/mL. After 2 h incubation at 37 °C, cells were lysed with a solution containing 50% (v:v) N,N-dimethylformamide and 20% (w:v) SDS, pH of 4.5, and then optical density was measured at 570 nm with a reference wavelength of 620 nm, using a microplate reader. MTT experiments have been performed in sestuplicate and repeated three times. Absorbance values were used to determine the IC_50_ using GraphPad Prism 5 Software (GraphPad Inc., San Diego, CA). Data are representative of three independent experiments; standard deviations (SD) were shown.

### Data analysis and statistical methods

Statistical significance between control and treated cells was analyzed by the means of GraphPad Prism 5.0 (GraphPad Software, Inc.; La Jolla, CA) software, using the analysis of variance (ANOVA) with Kruskal–Wallace test and post hoc Dunn’s Multiple Comparison Test. Significance was defined as *p < 0.05, **p < 0.01.

## Results and discussion

### Sericin extraction yield

Sericin extracted and isolated from silk can vary depending on extraction method and on source type. These two factors can highly influence the yield of extraction, as well as sericin molecular weight and its biological activity (Siritientong et al., 2016). White cocoons of *Bombyx mori* strain were boiled in hot water, at atmospheric pressure. Sericin was then isolated and purified according to the procedure described in the experimental session. The yield of extraction was calculated comparing the dry weight of sericin purified to the dry extract of the cocoon boiling water. The yield obtained ranged between 19% and 22%. Molecular weight and biological activity on healthy tissue were evaluated and reported in the following sections.

### Sericin–sunitinib conjugate synthesis

Sericin–sunitinib (SER–SUT) conjugate was synthesized using a click chemistry approach. The reaction involved the direct free radical grafting of SUT onto a biopolymeric chain, such as the protein sericin. According to synthetic procedure used for the achievement of the SER–SUT conjugate, a covalent bond has been introduced between the protein and the drug.

SER is a hydrophilic polypeptide characterized by fine water solubility as well as by low cytotoxicity towards living tissues. SER, indeed, has been successfully applied in various fields, especially in pharmaceutical and biomedical ones.

In the present work, in order to obtain the SER–SUT grafted protein, the initiation of the reaction occurred through the employment of hydrogen peroxide/l-ascorbic acid redox pair (Iemma et al., 2010). The high biocompatibility and water solubility of the initiating system allow the obtainment of the drug-conjugate in aqueous solutions, working at room temperature and avoiding the use of any organic solvent, which is not possible with conventional radical initiator systems (azo compounds and peroxides). Furthermore, non-toxic by-products are generated during the reaction.

The mechanism of the redox reaction is based on the oxidation of the l-ascorbic acid by H_2_O_2_ generating hydroxyl radical and l-ascorbate radical intermediates, responsible for reaction initiation. In particular, hydroxyl radical is one of the most reactive radicals among the reactive oxygen species, and it is able to generate SER macroradicals centered on polar aminoacids, on which labile hydrogens are abstracted (Song et al., 2006). The rate of hydrogen abstraction is dependent on the dissociation energy of the X–H bond to form the radical. The low molecular weight drug reacts with generated macroradicals and is grafted onto the SER biopolymeric chain.

The ratio 1:5 w/w between drug and protein was used for the grafting reaction.

**Figure 1.**
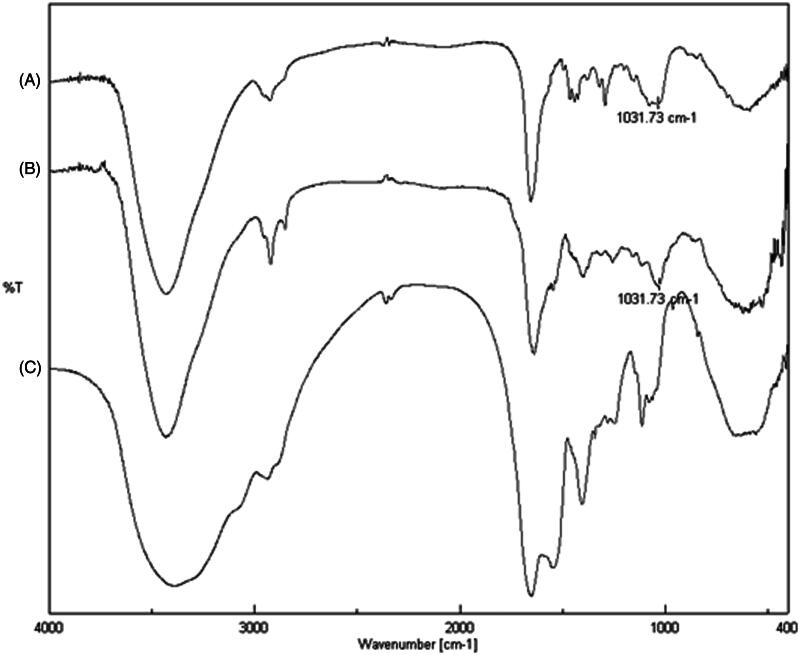
FT-IR spectra of (A) sunitinib malate, (B) sericin–sunitinib conjugate and (C) sericin.

FT-IR analyses were performed aiming at the identification and verification of the grafting of the drug on SER. Three different spectra were recorded (Figure 1): sunitinib malate (trace A); sericin–sunitinib conjugate (trace B) and sericin (trace C). The comparison of IR spectra showed the presence of a new signal at 1031.73 cm^−1^ in the spectrum of SER–SUT conjugate, that is identified also in the spectrum of sunitinib malate and corresponding to the stretching vibration of carbon–fluorine bond (stretching vibration of carbon–halogen single bond shows a typical band at ν < 1200 cm^−1^). On the contrary, the same peak is absent in the sericin spectrum.

For further evidence, UV/Vis spectra of sericin, sunitinb malate and sericin–sunitinib conjugate were also recorded between 250 nm and 700 nm (data not shown). Sericin and sunitinib malate showed typical absorbance at λ_max_ = 273 nm and λ_max_ = 426 nm, respectively. As confirmation of the occurred grafting, both peaks can be observed in the SER–SUT conjugate spectrum.

With the help of UV/Vis spectroscopy the amount of grafted SUT was determined, through the equation of the calibration curve of sunitinib malate. The content of SUT calculated was 6.5 mg of SUT per g of SER–SUT conjugate.

**Figure 2.**
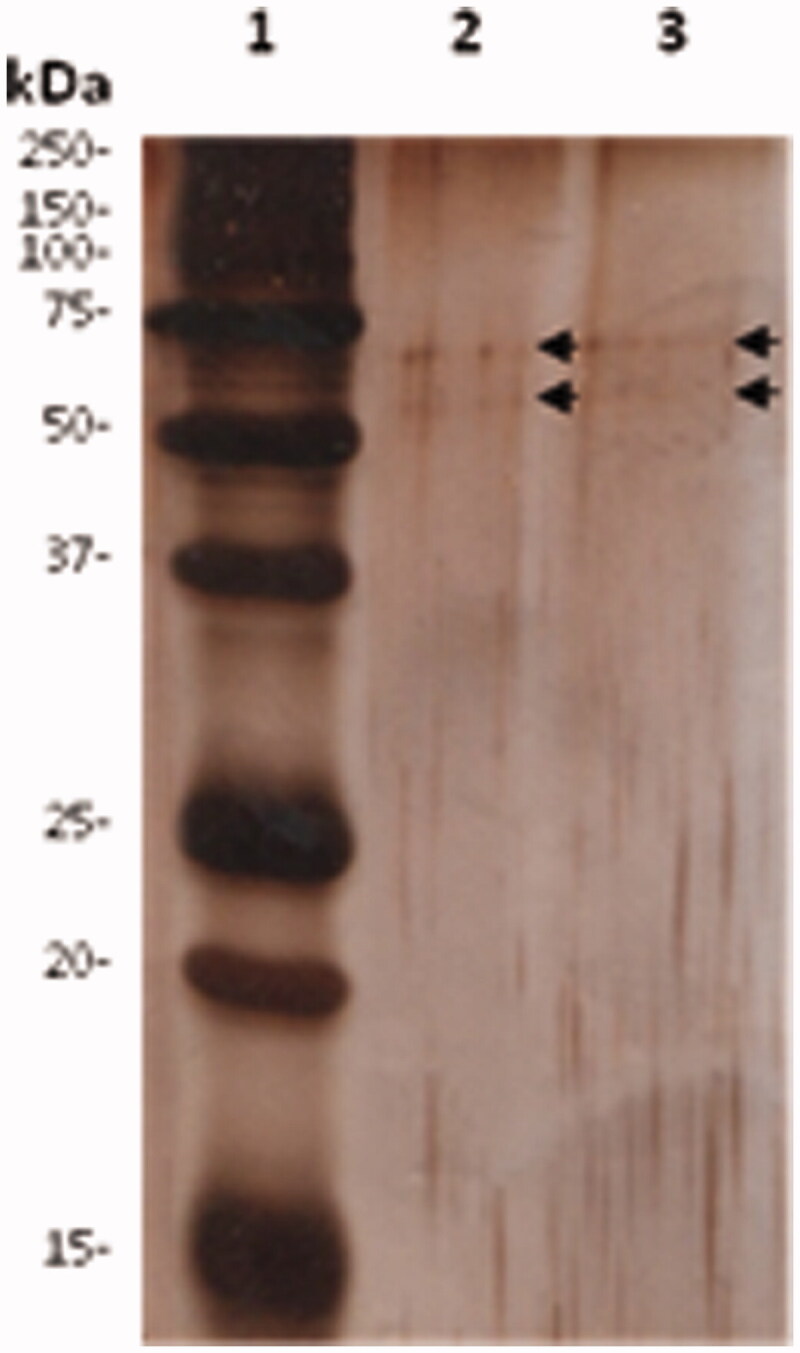
SDS-PAGE of SER and SER–SUT conjugate. 0.02 μg of proteins were separated by SDS-PAGE and stained with silver nitrate. Lane 1, marker; Lane 2, SER; Lane 3, SER–SUT conjugate. Black arrows indicate protein bands.

Literature data reported that sericin from silk may exist in different molecular weight forms, depending on the extraction processes, processing time, temperature or pH (Aramwit et al., 2011). The sericin forms involved in grafting process were evaluated by means of Sodium Dodecyl Sulphate-PolyAcrylamide Gel Electrophoresis (SDS-PAGE). Stock solutions of both SER and SER–SUT were prepared in loading buffer and 0.02 μg were analyzed. First, the aliquots were heated to 100 °C for 10 min, then loaded onto a 15% acrylamide gel (6% stacking gel and 15% resolving gel) and run under denaturing conditions. Electrophoresis was performed initially at 60 V until the samples migrated into the stacking gel, followed by 150 V until the last band of the marker reached the bottom of the gel. Immediately after the run, the gel was silver stained, in order to reveal protein bands. Only two faint protein bands with an apparent molecular weight ranging from 50 to 75 kDa were revealed by gel staining (Figure 2, black arrows), in both SER and SER–SUT conjugate, thus the grafting process did not produce any electrophoretic difference with respect to the original SER preformed biopolymeric chain.

### *In vitro* bioavailability studies

Many anticancer drugs, like sunitinib, suffer from low *in vivo* bioavailability. This drawback usually depends on several factors, such as low intrinsic activity, poor absorption, rapid metabolization and/or elimination. Solubility also plays a key role in determining drug oral bioavailability, since only the soluble fraction is considered bioaccessible (Bouayed et al., 2011). In order to evaluate the improvement of SER–SUT conjugate bioavailability, *in vitro* tests were performed. Resulting data were obtained using equation (1) and reported in Table 1 (see experimental section for more details).

(1) $$\mathrm{Bioavailability}\;(\%)=\frac{\mathrm{Sample}\;\mathrm{content}}{\mathrm{Total}\;\mathrm{content}}\times 100$$

**Table 1.** Bioavailability of sunitinib and SER–SUT conjugate.

Time points	Sutent® % bioavailability	SER–SUT conjugate % bioavailability
Phase I 2 h	20.3 ± 0.7	45.1 ± 0.9
Phase II 4 h	47.5 ± 0.8	28.8 ± 0.7
6 h	67.8 ± 1.0	73.9 ± 1.1

In 2011, Di Gion et al. (2011) have presented a work regarding several TKIs pharmacokinetic parameters. In that article, the reported absolute oral bioavailability percentage of sunitinib in humans, under fasted conditions, was around 50%.

In our study, the bioavailability evaluation was performed comparing formulated sunitinib, also commercially known as Sutent® (*Pfizer*) and SER–SUT conjugate. Data reported shows a good bioavailability percentage after six hours for both samples, slightly improved in the case of the conjugate. However, a significative increase is yet observable after 2 h, meaning a ready dissolution of the sericin protein–sunitinib conjugate in the gastric environment.

### *In vitro* membrane permeability studies

The ability of SER–SUT conjugate to cross biological membranes compared with free SUT has been investigated through permeability studies, using phosphatidylcholine liposomes.

It is well-known that drug’s therapeutic and cytotoxic activities, as well as absorption, distribution, metabolism and excretion, are governed by drug affinity with biological membranes. Interactions between drug and cell membrane are, indeed, responsible for its bioaccumulation. The entire drug life *in vivo* is influenced by its interaction with membranes and often the therapeutic target is within the membrane itself, as in the case of SUT.

Bioaccessibility and bioconcentration is strongly dependent on the ability of the drug to cross the phospholipidic bilayer *via* diffusion, thus on drug hydrophilic/hydrophobic ratio, which can be quantitatively expressed as partition coefficient between two phases. A simple model widely used to determine drugs partition coefficient is based on a *n*-octanol/water system that is thermodynamically different from biological models and it is considered only an approximation of the physiological condition.

However, liposomal model is a more reliable system, able to mimic the cell environment. Liposomes are closed membranes made of amphiphilic lipids, characterized by a liquid crystal structure, that take into account of the surface charges involved in the drug/cell electrostatic interactions, particularly important for charged and polar drugs. In addition, the complexity of the bilayer structure and steric forces can contribute, either positively or negatively, in drugs bioaccessibility. For such reasons, the partition coefficient obtained by anisotropic membrane/buffer solution system better predicts drugs behavior in biological environment than an isotropic 2-phase solvent system (i.e. *n*-octanol/water).

**Figure 3.**
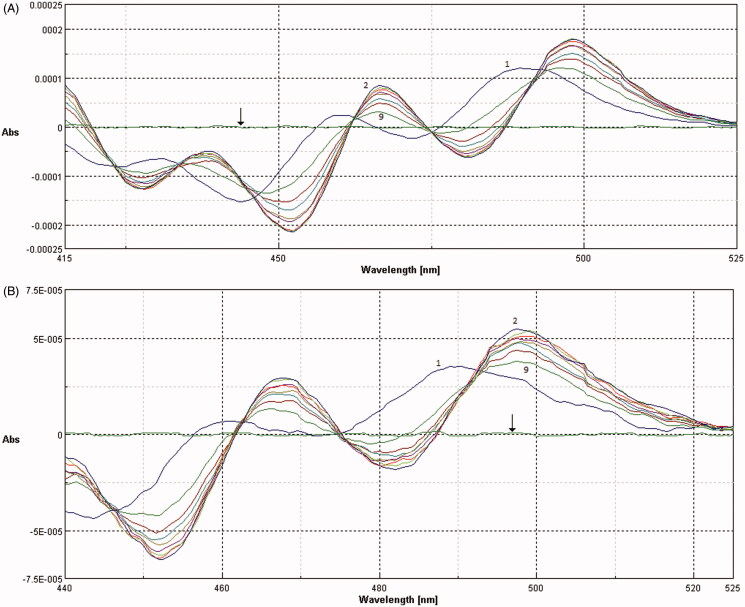
Second derivative spectra of SUT (A) and SER–SUT conjugate (B) calculated from the absorption spectra, at different concentration of SUVs (1) 0 μM, (2) 150 μM, (3) 300 μM, (4) 450 μM, (5) 600 μM, (6) 750 μM, (7) 900 μM, (8) 1050 μM, (9) 1200 μM. Black arrows indicate the second derivative absorption spectra of SUVs in absence of SUT or SER–SUT.

Analytical determination of membrane/buffer solution partition coefficient by spectrophotometry requires phase separation, which disturbs the established equilibrium state. The interruption of the equilibrium can cause inaccuracy of the measurement, as well as loss of the bilayer drug content. Furthermore, liposome suspensions cause interference in absorption spectra, especially in UV region, because of the intense background signal due to the light scattered by lipid vesicles. However, investigation by second derivative spectroscopic methodology can avoid vesicles interference, without disturbing partition equilibrium, improving at the same time the resolution of overlapped bands. Moreover, SUT absorbs in the visible region, where interference is completely eliminated in second derivative spectroscopy (Figure 3, black arrows).

Since the sunitinib peak is observable also in SER–SUT conjugate absorption spectrum, the same method could be applied for the determination of bioconjugate partition coefficient.

For the experimental procedure small unilamellar vesicles (SUVs) were produced *via* self-assembly of phosphatidylcholine (PC). SUVs size was measured by DLS analysis showing a homogeneous population of liposomes with a mean diameter in the range of 170–180 nm. The absorption spectra of 20 μM SUT and SER–SUT conjugate with equal SUT concentration, recorded in suspensions containing various concentrations of PC liposomes, are shown in Figure 3.

The presence of isosbestic points in the overlaid spectra at different concentrations of PC confirmed the existence of an equilibrium between drug dissolved in the polar aqueous phase of the buffer solution and in the non-polar PC bilayer phase.

The membrane/buffer solution partition coefficient (Kp) of both SUT and SER–SUT conjugate were calculated using the following Equation (2) and applying the Scott-plot method:

(2) $$\Delta D=\frac{\mathrm{Kp}\;\Delta D_{\max}[L]}{\left[W\right]+\mathrm{Kp}[L]}$$

where ΔD is the derivative intensity difference of drug before and after the addition of EPC SUV, [L] and [W] are the concentrations of EPC and water, respectively, and ΔD_max_ is the maximum ΔD value assuming all drug is partitioned in PC SUVs. Partition coefficient was expressed as log(Kp) for a ready interpretation of the data. Results obtained are reported in Table 2 and expressed as mean value ± standard deviation.

**Table 2.** Log(Kp) and IC_50_ values of sunitinib and SER–SUT conjugate.

		IC_50_ (μM)	
Compound	Log(Kp)	MCF-7	MCF-10A
Sunitinib	3.4 ± 0.2	24.6 ± 1.2	>76
Sericin–sunitinib conjugate	4.1 ± 0.3	7.8 ± 0.9	>76

According to the Lipinski’s “rule of five”, one criterion for a good oral bioavailability is that the log of the partition coefficient, log(Kp) or the log of the ratio of the solubility of the drug in *n*-octanol/water, should be less than 5. From the scientific literature, we know that log(Kp)_o/w_ for sunitinib is 5.2 (Roskoski, 2007). However, in a more complex model, such as membrane/buffer solution system, log(Kp) value take into account surface charges, electrostatic interactions and steric forces. Furthermore, the partitioning of the solutes within lipidic membranes occurs by a mechanism which is different from the one occurring in oil phase.

Using this model, SUT showed a good log(Kp) value (see Table 2), while SER–SUT conjugate partition coefficient resulted augmented. In particular, this latter behavior can be likely ascribed to the protein structure that confers plasticity and adaptability to the lipid bilayer.

### Cell viability assay

**Figure 4.**
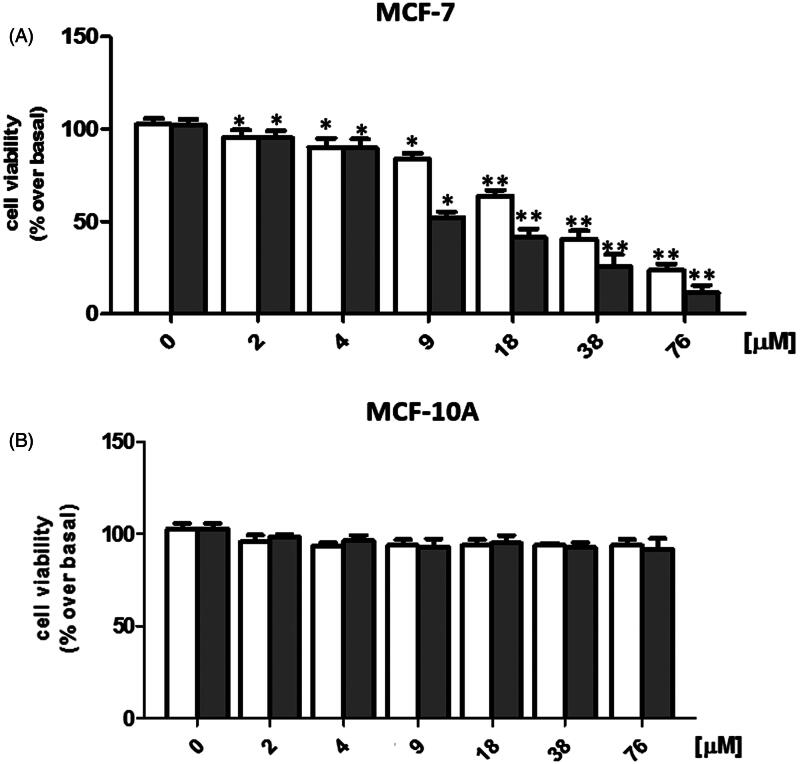
Graphs of MCF-7 (A) and MCF-10A (B) cell viability, exposed to □Sunitinib; ▪sericin–sunitinib conjugate. *p < 0.05; **p < 0.01.

In order to evaluate the antitumor activity of SER–SUT conjugate, estrogen receptor positive (ER+) MCF-7 cells were used. Concentrations of SER–SUT conjugate have been chosen taking into account the content of SUT conjugated to SER from UV/Vis experiments (see “sericin–sunitinib conjugate synthesis” paragraph), so that same concentrations of SUT alone and SER–SUT conjugate were tested. Cells were grown up to 50–60% confluence and treated with increasing doses of both SUT and SER–SUT conjugate (see experimental procedures for details) and, after 72 h, cell viability was determined by MTT assay (Iacopetta et al., 2017). Both SUT and SER–SUT exhibited a dose-dependent reduction of MCF-7 cells viability at the end of the experiment (Figure 4, panel A), whereas no effect has been noticed on the normal MCF-10A cells viability (Figure 4, panel B). IC_50_ values, reported in Table 2 and calculated for both SUT and SER–SUT in MCF-7 and MCF-10A cells, demonstrated an increase of SER–SUT conjugate cytotoxic effect, which is about three-times higher than that of SUT, in MCF-7 cells. On the contrary, neither SUT nor SER–SUT affect MCF-10A cells viability (IC_50 _>_ _76 μM, Table 2).

The main concern for using SER for biomedical applications is its allergic property, hence its safety. This aspect has been recently discussed in a work of Ampawong et al., in which SER showed a tolerogenic activity (Ampawong & Aramwit, 2016). Moreover, at the same concentrations and conditions used in our experiments, SER alone did not exert any toxic effect on MCF-7 and MCF-10A cells (data not shown). These outcomes suggest that the increased antitumor activity might be due to the improved solubility and cell permeability of SER–SUT conjugate.

## Conclusions

In the present research paper, we have reported the free radical grafting of sunitinib onto sericin polypeptidic chain, *via* single-step click reaction. The biocompatible and water soluble redox couple (l-ascorbic acid/hydrogen peroxide) has been employed in order to initiate the reaction. The covalent attachment of sunitinib has been obtained by hydroxyl radical action generated during the reaction. It was confirmed by FT-IR and UV/Vis spectroscopy, after protein purification.

*In vitro* gastrointestinal bioavailability experiments have highlighted an improved percentage of sunitinib that can cross the simulated GI lumen, compared with the commercially available drug (Sutent®). This effect is certainly due to augmented water solubility of the sericin–sunitinib conjugate and it is evident from the very first two hours, in which we observed the ready dissolution of the functionalized material.

Second derivative spectroscopy has given the opportunity to evaluate the interaction between sericin–sunitinib conjugates and cell membrane models, such as phosphatidylcholine small unilamellar vesicles. The study demonstrated an improvement of sericin–sunitinib membrane permeability, expressed as an increase of membrane/buffer solution partition coefficient.

In the end, the *in vitro* cytotoxicity tests showed that the newly synthesized sericin–sunitinib conjugate is three times more effective than free sunitinib, thanks to the characteristics acquired by conjugation with the protein.

Thanks to these results, we can state that the production of a sericin-grafted biomaterial is a valuable method to improve the bioavailability and permeability of drugs like sunitinib.

## References

[CIT0001] Akash MSH, Rehman K, Parveen A, et al. (2016). Antibody-drug conjugates as drug carrier systems for bioactive agents. Int J Polym Mater Polym Biomater 65:1–10

[CIT0002] Alley SC, Okeley NM, Senter PD. (2010). Antibody-drug conjugates: targeted drug delivery for cancer. Curr Opin Chem Biol 14:529–37 20643572 10.1016/j.cbpa.2010.06.170

[CIT0003] Amidon GL, Lennernäs H, Shah VP, et al. (1995). A theoretical basis for a biopharmaceutic drug classification: the correlation of in vitro drug product dissolution and in vivo bioavailability. Pharm Res 12:413–20 7617530 10.1023/a:1016212804288

[CIT0004] Ampawong S, Aramwit P. (2016). Tolerogenic responses of CD206+, CD83+, FOXP3+, and CTLA-4 to sericin/polyvinyl alcohol/glycerin scaffolds relevant to IL-33 and HSP60 activity. Histol Histopathol 31:1011–27 26864661 10.14670/HH-11-733

[CIT0005] Aramwit P, Siritientong T, Srichana T. (2011). Potential applications of silk sericin, a natural protein from textile industry by-products. Waste Manag Res 30:217–24 21558082 10.1177/0734242X11404733

[CIT0006] Bouayed J, Hoffmann L, Bohn T. (2011). Total phenolics, flavonoids, anthocyanins and antioxidant activity following simulated gastro-intestinal digestion and dialysis of apple varieties: bioaccessibility and potential uptake. Food Chem 128:14–21 25214323 10.1016/j.foodchem.2011.02.052

[CIT0007] Coburn JM, Kaplan DL. (2015). Engineering biomaterial-drug conjugates for local and sustained chemotherapeutic delivery. Bioconjug Chem 26:1212–23 25689115 10.1021/acs.bioconjchem.5b00046 PMC4856894

[CIT0008] Deng L, Zhang H, Yang M, et al. (2014). Improving properties of superabsorbent composite induced by using alkaline protease hydrolyzed‐sericin (APh‐sericin). Polymer Compos 35:509–15

[CIT0009] Di Gion P, Kanefendt F, Lindauer A, et al. (2011). Clinical pharmacokinetics of tyrosine kinase inhibitors: focus on pyrimidines, pyridines and pyrroles. Clin Pharmacokinet 50:551–603 21827214 10.2165/11593320-000000000-00000

[CIT0010] Etrych T, Šubr V, Strohalm J, et al. (2012). HPMA copolymer-doxorubicin conjugates: the effects of molecular weight and architecture on biodistribution and in vivo activity. J Control Release 164:346–54 22759979 10.1016/j.jconrel.2012.06.029

[CIT0011] Fattahi A, Asgarshamsi M, Hasanzadeh F, et al. (2015). Methotrexate-grafted-oligochitosan micelles as drug carriers: synthesis and biological evaluations. J Mater Sci Mater Med 26:1–10 10.1007/s10856-015-5407-5 25677115

[CIT0012] Grande F, Parisi OI, Mordocco RA, et al. (2016). Quercetin derivatives as novel antihypertensive agents: synthesis and physiological characterization. Eur J Pharm Sci 82:161–70 26631584 10.1016/j.ejps.2015.11.021

[CIT0013] Herbrink M, Nuijen B, Schellens JH, et al. (2015). Variability in bioavailability of small molecular tyrosine kinase inhibitors. Cancer Treat Rev 41:412–22 25818541 10.1016/j.ctrv.2015.03.005

[CIT0014] Iacopetta D, Rosano C, Puoci F, et al. (2017). Multifaceted properties of 1,4-dimethylcarbazoles: focus on trimethoxybenzamide and trimethoxyphenylurea derivatives as novel human topoisomerase II inhibitors. Eur J Pharm Sci 96:263–72 27702608 10.1016/j.ejps.2016.09.039

[CIT0015] Iemma F, Puoci F, Curcio M, et al. (2010). Ferulic acid as a comonomer in the synthesis of a novel polymeric chain with biological properties. J Appl Polym Sci 115:784–9

[CIT0016] Izzedine H, Buhaescu I, Rixe O, et al. (2007). Sunitinib malate. Cancer Chemother Pharmacol 60:357–64 17136543 10.1007/s00280-006-0376-5

[CIT0017] Jabbour E, Kantarjian H, Cortes J. (2015). Use of second- and third-generation tyrosine kinase inhibitors in the treatment of chronic myeloid leukemia: an evolving treatment paradigm. Clin Lymphoma Myeloma Leuk 15:323–34 25971713 10.1016/j.clml.2015.03.006 PMC5141582

[CIT0018] Kassem MG, Motiur Rahman AFM, Korashy HM. 2012. Chapter 9 – Sunitinib malate. In: Harry GB, ed. Profiles of drug substances, excipients and related methodology. Cambridge, MA: Academic Press, 363–88 10.1016/B978-0-12-397220-0.00009-X 22469323

[CIT0019] Kok R, Grijpstra F, Nederhoed K, et al. (1999). Renal drug delivery with low-molecular-weight proteins: the effect of charge modifications on the body distribution of drug-lysozyme conjugates. Drug Deliv 6:1–8

[CIT0020] Laemmli UK. (1970). Cleavage of structural proteins during the assembly of the head of bacteriophage T4. Nature 227:680–5 5432063 10.1038/227680a0

[CIT0021] Lamboni L, Gauthier M, Yang G, et al. (2015). Silk sericin: a versatile material for tissue engineering and drug delivery. Biotechnol Adv 33:1855–67 26523781 10.1016/j.biotechadv.2015.10.014

[CIT0022] Liu B, Song Y-W, Jin L, et al. (2015). Silk structure and degradation. Colloids Surf B Biointerfaces 131:122–8 25982316 10.1016/j.colsurfb.2015.04.040

[CIT0023] Madeo M, Carrisi C, Iacopetta D, et al. (2009). Abundant expression and purification of biologically active mitochondrial citrate carrier in baculovirus-infected insect cells. J Bioenerg Biomembr 41:289–97 19629661 10.1007/s10863-009-9226-6

[CIT0024] Moscho A, Orwar O, Chiu DT, et al. (1996). Rapid preparation of giant unilamellar vesicles. Proc Natl Acad Sci USA 93:11443–7 8876154 10.1073/pnas.93.21.11443 PMC38076

[CIT0025] Napavichayanun S, Amornsudthiwat P, Pienpinijtham P, et al. (2015). Interaction and effectiveness of antimicrobials along with healing-promoting agents in a novel biocellulose wound dressing. Mater Sci Eng C 55:95–104 10.1016/j.msec.2015.05.026 26117743

[CIT0026] Padamwar M, Pawar A. (2004). Silk sericin and its applications: a review. J Sci Ind Res 63:323–9

[CIT0027] Pang X, Du H-L, Zhang H-Q, et al. (2013). Polymer-drug conjugates: present state of play and future perspectives. Drug Discov Today 18:1316–22 24055841 10.1016/j.drudis.2013.09.007

[CIT0028] Papaetis GS, Syrigos KN. (2009). Sunitinib: a multitargeted receptor tyrosine kinase inhibitor in the era of molecular cancer therapies. BioDrugs 23:377–89 19894779 10.2165/11318860-000000000-00000

[CIT0029] Parisi OI, Fiorillo M, Scrivano L, et al. (2015a). Sericin/poly (ethylcyanoacrylate) nanospheres by interfacial polymerization for enhanced bioefficacy of fenofibrate: in vitro and in vivo studies. Biomacromolecules 16:3126–33 26348208 10.1021/acs.biomac.5b00746

[CIT0030] Parisi OI, Morelli C, Scrivano L, et al. (2015b). Controlled release of sunitinib in targeted cancer therapy: smart magnetically responsive hydrogels as restricted access materials. RSC Adv 5:65308–15

[CIT0031] Puoci F, Morelli C, Cirillo G, et al. (2012). Anticancer activity of a quercetin-based polymer towards HeLa cancer cells. Anticancer Res 32:2843–7 22753746

[CIT0032] Roskoski R. (2007). Sunitinib: a VEGF and PDGF receptor protein kinase and angiogenesis inhibitor. Biochem Biophys Res Commun 356:323–8 17367763 10.1016/j.bbrc.2007.02.156

[CIT0033] Saturnino C, Sinicropi MS, Parisi OI, et al. (2014). Acetylated hyaluronic acid: enhanced bioavailability and biological studies. BioMed Res Int 2014:1–7 10.1155/2014/921549 PMC4121155 25114930

[CIT0034] Savitzky A, Golay MJ. (1964). Smoothing and differentiation of data by simplified least squares procedures. Anal Chem 36:1627–39 10.1021/ac60319a045 22324618

[CIT0035] Siritientong T, Bonani W, Motta A, et al. (2016). The effects of Bombyx mori silk strain and extraction time on the molecular and biological characteristics of sericin. Biosci Biotechnol Biochem 80:241–9 26399155 10.1080/09168451.2015.1088375

[CIT0036] Song Y, Jin Y, Sun J, et al. (2006). Graft copolymerization of methyl acrylate onto silk sericin initiated by tert‐butyl hydroperoxide. Polym Int 55:1350–4

[CIT0037] Takegami S, Kitamura K, Ohsugi M, et al. (2015). Partitioning of organophosphorus pesticides into phosphatidylcholine small unilamellar vesicles studied by second-derivative spectrophotometry. Spectrochim Acta A Mol Biomol Spectros 145:198–202 10.1016/j.saa.2015.02.061 25775945

[CIT0038] Vicent MJ. (2007). Polymer-drug conjugates as modulators of cellular apoptosis. AAPS J 9:E200–07 17907762 10.1208/aapsj0902022 PMC2751409

[CIT0039] Vittorio O, Cirillo G, Iemma F, et al. (2012). Dextran-catechin conjugate: a potential treatment against the pancreatic ductal adenocarcinoma. Pharm Res 29:2601–14 22622510 10.1007/s11095-012-0790-9

[CIT0040] Walde P, Cosentino K, Engel H, et al. (2010). Giant vesicles: preparations and applications. ChemBioChem 11:848–65 20336703 10.1002/cbic.201000010

[CIT0041] Wu L, Zhang Z, Yao H, et al. (2014). Clinical efficacy of second-generation tyrosine kinase inhibitors in imatinib-resistant gastrointestinal stromal tumors: a meta-analysis of recent clinical trials. Drug Des Dev Ther 8:2061 10.2147/DDDT.S63840 PMC4219427 25378911

[CIT0042] Zhang Y-Q, Ma Y, Xia Y-Y, et al. (2006a). Silk sericin–insulin bioconjugates: Synthesis, characterization and biological activity. J Control Release 115:307–15 17034892 10.1016/j.jconrel.2006.08.019

[CIT0043] Zhang YQ, Tao ML, Shen WD, et al. (2006b). Synthesis of silk sericin peptides–L‐asparaginase bioconjugates and their characterization. J Chem Technol Biotechnol 81:136–45

[CIT0044] Zhao L, Liu C, Sun Y, et al. (2012). A rapid and simplified method for protein silver staining in polyacrylamide gels. Electrophoresis 33:2143–4 22821490 10.1002/elps.201200107

